# Activity (transcription) of the genes for MLH1, MSH2 and p53 in sporadic colorectal tumours with micro-satellite instability

**DOI:** 10.1038/sj.bjc.6601823

**Published:** 2004-04-20

**Authors:** S I H Tou, E R Drye, P B Boulos, S J Hollingsworth

**Affiliations:** 1 Colorectal Unit, Department of Surgery, Royal Free and University College Medical School, Charles Bell House, 67-73 Riding House Street, London W1W 7EJ, UK

**Keywords:** micro-satellite instability, MLH1, MSH2, p53, mismatch repair, gene transcription

## Abstract

Micro-satellite instability (MSI) is relevant in the management of colorectal cancers (CRC) and relies on analysis of gene mutations, or production of the proteins involved in DNA mismatch repair (e.g. MLH1, MSH2). p53 mutation is also relevant in MSI, but high-level CRC (MSI-H) demonstrate fewer mutations than low-level (MSI-L) or stable (MSS) cancers. Recently, the importance of gene activity (transcription) in MSI has been identified, where rather than being mutated genes have been downregulated. In this study, 67 sporadic CRC and eight samples of normal bowel were analysed for MSI status (by SSCP) and levels of MLH1, MSH2 and p53 gene transcription (by RT–PCR and scanning densitometry). Micro-satellite instability correlated with gender and site, with more MSI-H CRC in females (*P*<0.02) and in the right colon (*P*<0.04). In MSI-H, p53 transcription was markedly reduced (*P*<0.003). Compared to normal bowel, MLH1 transcription was elevated in all cancers (*P*<0.01), while MSH2 transcription was elevated only in MSI-H (*P*<0.04). There was a direct correlation between MLH1 and MSH2 transcription (*P*<0.001). Although fewer mutations are reported in MSI-H than MSI-L/MSS, these results suggest that reduced p53 transcription might account for decreased tumour suppression in MSI-H. The direct correlation between MLH1 and MSH2 transcription suggests that control of these genes might be coordinated.

Colorectal cancers (CRC) exhibit two distinct forms of genomic instability believed to underlie their development. Chromosomal instability (CIN) is associated with loss of function of the tumour-suppressor gene p53 (Lengauer *et al*, 1997) and hence the G1-to-S phase checkpoint of the cell cycle, resulting in ‘accumulation’ of mutations, leading to tumorigenesis. In contrast, micro-satellite instability (MSI) results from loss of function of the mismatch repair (MMR) genes, principally MLH1 and MSH2 (Papadopouos and Lindblom, 1997). With normal functioning MMR genes, errors in DNA replication are identified, the ‘mutated’ sequences excised and the correct sequence re-synthesised. However, loss of MMR leads to an accumulation of errors within micro-satellite regions, resulting in the disturbance of regulation and/or activity of a number of genes such as TGF*β*RII (Parsons *et al*, 1995) or TCF-4 (Duval *et al*, 1999), again leading to tumorigenesis.

Micro-satellite instability occurs in almost all hereditary nonpolyposis CRC (HNPCC; Aaltonen *et al*, 1993), which accounts for <1% of CRC overall (Percesepe *et al*, 2001; Samowitz *et al*, 2001) and approximately 15% of sporadic CRC (Kim *et al*, 1994; Feeley *et al*, 1999; Gryfe *et al*, 2000; Wright *et al*, 2000). In the National Cancer Institute (NCI) guidelines for the detection of MSI (Boland *et al*, 1998), a panel of micro-satellite loci containing two mono-nucleotide repeats (BAT25 and BAT26) and three di-nucleotide repeats (D2S123, D5S346 and D17S250) are investigated. Cancers are then subdivided into three groups; high-level MSI (MSI-H) that demonstrate instability in >30–40% of loci, low-level MSI (MSI-L) instability of <30–40% of loci, and stable cancers (MSS) with no instability, that is, all loci normal.

When more (than the five recommended) micro-satellite regions are examined, almost all CRCs demonstrate some degree of MSI (usually MSI-L; Laiho *et al*, 2002). As a result, there is controversy as to whether MSS and MSI-L cancers are distinctly separate or are similar. Jass *et al* (2002), among others, favour a clear and distinct separation between these groups, as the identification of ‘milder mutator’ phenotype MSI-L cancers can be of clinical significance. MSI-H cancers have distinctive clinical features such as large tumour size, proximal predominance, poor histological differentiation, mucinous growth and lymphocytic infiltration, with better prognosis compared to stage-matched MSS cancers (Lothe *et al*, 1993; Kim *et al*, 1994; Boland *et al*, 1998; Gryfe *et al*, 2000).

Although mutations in MMR genes are believed to account for most HNPCC, the mechanism responsible for the MSI-H phenotype in sporadic CRC seems to involve methylation of MMR gene sequences, resulting in downregulation of gene expression (transcription) and subsequently less functional protein (Kane *et al*, 1997; Cunningham *et al*, 1998; Herman *et al*, 1998; Kuismanen *et al*, 1999). Since overexpression of MLH1 and MSH2 induces apoptosis (Zhang *et al*, 1999), the relationships between these genes and the mutation and function of the tumour-suppressor gene p53 have been of interest. There appears to be an inverse relationship between MSI ‘severity’ (status) and p53 mutation (Cottu *et al*, 1996; Breivik *et al*, 1997; Olschwang *et al*, 1997; Forster *et al*, 1998; Iacopetta *et al*, 1998), which seems paradoxical as a greater rate of p53 mutation in higher ‘mutator phenotype’ MSI-H cancers might be expected. The mechanism underlying this effect is unclear but might relate to the degree of DNA methylation and consequently gene activity in the MMR genes (Cunningham *et al*, 1998; Kuismanen *et al*, 1999), although this has not been described.

Numerous studies have investigated the relationship between MLH1, MSH2 and p53 in MSI, by examining principally mutations in the gene DNA sequences, or production of the relevant protein. Few if any studies, however, have examined gene activity, that is, gene transcription, which has particular relevance in understanding the control of gene regulation in the process(es) of tumour development and progression. Therefore in this study, the activity (transcription) of MLH1, MSH2 and p53 in sporadic CRC with MSI is investigated by reverse transcription–polymerase chain reaction (RT–PCR) analysis of total cellular RNA extracted from archival wax-embedded samples.

## MATERIALS AND METHODS

### Sample collection

The study was performed after approval by the Joint University College London/University College Hospitals London Committees on the Ethics of Human Research. There were 67 paraffin-embedded cancer archival samples from 67 patients who had undergone colonic resection and had been followed up for at least 7 years with no evidence of metachronus cancers. Normal control samples of colon were obtained from eight patients undergoing colonic resection, seven for cancer and one for a large tubullo-villous adenoma, and were paraffin-embedded. Gender, age, cancer site and Dukes' classification were recorded. Cancers were defined as right-sided when proximal, and left-sided when distal, to the splenic flexure.

### Analysis of MSI

Micro-satellite instability status was determined by PCR-SSCP (single-stranded conformational polymorphism; Sengupta *et al*, 1997), using a panel of six micro-satellite loci, three mono-nucleotide (BAT25, BAT26, BAT40) and three di-nucleotide repeat regions (D5S346, D2S123 and D17S250; Boland *et al*, 1998).

### Analysis of gene transcription

Total cellular RNA was extracted from paraffin-embedded samples using the commercially available Ambion® RNA isolation kit (Ambion® Ltd; Cambridgeshire, UK). Samples were prepared as follows: two 20 *μ*m paraffin-embedded sections were de-paraffinised in xylene (BDH®; Merck, UK), washed with 100% ethanol and then dried for 5 min in air (at room temperature). Tissue was digested by treatment with proteinase K (1 mg ml^−1^) for 2 h at 45°C and then solubilised in 600 *μ*l guanidinium-based RNA extraction buffer. Following extraction with 700 *μ*l acid phenol : chloroform, RNA was precipitated in isopropanol (BDH®; Merck, UK) using linear acrylamide as a carrier. The precipitated RNA was washed in 75% ethanol, re-dissolved in 10 *μ*l RNA Storage Solution™ and then stored at −20°C. Complimentary DNA (cDNA) was produced from 4 *μ*g of total cellular RNA using MMLV-RT (Sigma®, UK) and random hexamers (New England BioLabs® Inc, USA) according to a standard protocol. Subsequent PCR reactions were performed on 600 ng of cDNA, amplified in a 50 *μ*l reaction using DNA polymerase REDTaq™ (Sigma®, UK) in a Techne Genius Thermocycler (Jencons PLS, UK). A standard ‘hot start’ PCR was used in conjunction with cycles of increasing stringency, to a total of 40 cycles of amplification as follows: 94°C (45 s), 50°C (45 s), 72°C (45 s) for 10 cycles, then 94°C (45 s), 55°C (45 s), 72°C (45 s) for 10 cycles and then 94°C (45 s), 58°C (45 s), 72°C (45 s) for a further 20 cycles.

For the amplification of test genes, sets of primers were designed specifically to have small amplicon sizes (to maximise the yield), and which spanned at least one exon–exon boundary to avoid the aberrant amplification of any contaminating DNA sequences (Masuda *et al*, 1999). Furthermore, to ensure that for each test gene analysed the sequences amplified were reflective of processed and full-length messenger RNA, two sets of primers were utilised (in separate PCR reactions), designed to amplify sequences towards the 5′ and separately, the 3′ regions of each gene. The primer sequences used are presented in Table 1

**Table 1:** Primer sequences

**Gene**	**Region**	**Primer sequence**	**Direction**	**Size (bp)**
MLH1	5′	-TGTTAAAGAGGGAGGCCTGA-	Sense	92
		-TGAACCTTTCACATACAATATCCA-	Antisense	
				
	3′	-AAGAAGGCTGAGATGCTTGC-	Sense	102
		-CAAAGGGGGCACATAGTTGT-	Antisense	
				
MSH2	5′	-AAGGCATCCAAGGAGAATGA-	Sense	106
		-TGGAAGCTGACATATCATTGTTAC-	Antisense	
				
	3′	-GAGTCTCCACGTTCATTGGCT-	Sense	103
		-GAAGTTCCTCTTCCCAATTCA-	Antisense	
				
p53	5′	-CCCCTCTGAGTCAGGAAACA-	Sense	93
		-AATCATCCATTGCTTGGGAC-	Antisense	
				
	3′	-AGGCCTTGGAACTCAAGGAT-	Sense	106
		-TTATGGCGGGAGGTAGACTG-	Antisense	
				
GAP-3	—	-GAGTCAACGGATTTGGTCGT-	Sense	103
		-TTGAGGTCAATGAAGGGGTC-	Antisense	

Primer sequences used for RT–PCR analyses.

. PCR products were resolved on 2% agarose (BDH®, Merck, UK), and transcripts analysed by scanning densitometry (measured as total integrated optical density, IOD) using LabWorks Imaging and Acquisition software (UVP, Cambridge, UK). Levels of gene transcription were determined from the ratio of test gene PCR band IOD and that of its internal control house-keeping gene glycerylaldehyde 3-dehydrogenase (GAP-3). Samples were included for analysis only when there was consensus between levels of transcription in transcripts amplified from both 5′ and 3′ regions. Consensus IOD ratios were then averaged. For all samples, two separate PCR reactions were performed with the mean IOD ratio used for further analysis.

In order to investigate the transcription of MLH1, MSH2 and p53 further, samples were analysed according to the presence of mutations in particular markers, as follows: (1) no mutations (MSS cancers), (2) mutations present only in mono-nucleotide markers (di-nucleotide markers normal), (3) mutations present only in di-nucleotide markers (mono-nucleotide markers normal) and (4) mutations present in both mono- and di-nucleotide markers.

### Statistics

The results were summarised using descriptive statistics and comparisons between group ages made by Mann–Whitney *U* test. The MSI status and Dukes' staging, gender or site, or between Dukes' staging with gender or site, were compared by *χ*^2^ test. The levels of gene transcription are presented as mean values with s.e.m. (in parentheses) following analysis for uniformity and skewness, and by one-sample Kolmogorov–Smirnov test for normality. Comparisons between groups were made by one-way analysis of variance (ANOVA) with Bonferroni's correction to identify differences between individual group means; all tests were two-tailed. Correlation between factors was analysed by Pearson's bivariate correlation analysis. Statistical significance was accepted at *P*<0.05; analyses yielding nonstatistically significant results are not cited.

## RESULTS

A total of 67 specimens (median age 73 (range 38–100) years) from 35 males (70 (38–100) years) and 32 females (77 (53–91) years) with CRC, and eight specimens of normal bowel (68.5 (45–87) years) from five males (66 (57–82) years) and three females (71 (45–87) years) were obtained. The age and sex distribution was comparable for CRC (*P*>0.1) and normal samples (*P*>0.1). Of the 67 CRC examined, the majority were MSS (55%; *P*<0.001, *χ*^2^, Table 2

**Table 2:** Demographics

	**Male**	**Female**	**Total**
*Site*			
Left colon	21 (54%)	18 (46%)	39 (58%)
Right colon	14 (50%)	14 (50%)	28 (42%)
			
*Dukes' stage*			
A	4 (67%)	2 (33%)	6 (9%)
B	17 (53%)	15 (47%)	32 (48%)^†^
C	12 (50%)	12 (50%)	24 (36%)^†^
D	2 (40%)	3 (60%)	5 (7%)
			
*Micro-satellite status*			
MSS	21 (57%)	16 (43%)	37 (55%)^†^
MSI-L	12 (67%)^‡^	6 (33%)	18 (27%)
MSI-H	2 (17%)	10 (83%)^‡^	12 (18%)

Demographic information, micro-satellite status, site of tumour and Dukes' stage for sporadic CRC analysed. Percentages in parentheses represent the percentage/gender per MSI, site or Dukes' stage group. Values of significance: ^†^*P*<0.001 and ^‡^*P*<0.02, both by *χ*^2^ test.

), with fewer MSI-L (29%) and MSI-H (18%) cases. These were more left-sided cancers (58%; although not significant) and were mostly Dukes' B (48%) or C (36%) stage (*P*<0.001, *χ*^2^, Table 2).

Dukes' stage distribution was similar in both sexes (Table 2). While Dukes' stage B and C cancers were of a similar distribution in the left and right sides of the colon (Table 3

**Table 3:** Tumour site

	**Left colon**	**Right colon**	**Total**
*Micro-satellite status*			
MSS	24 (65%)	13 (35%)	37 (55%)
MSI-L	12 (67%)	6 (33%)	18 (27%)
MSI-H	3 (25%)	9 (75%)^†^	12 (18%)
			
*Dukes' stage*			
A	6 (100%)^‡^	0 (−)	6 (9%)
B	17 (53%)	15 (47%)	32 (48%)
C	11 (46%)	13 (54%)	24 (36%)
D	5 (100%)^‡^	0 (−)	5 (7%)

Site of tumour with micro-satellite status and Dukes' stage for CRC analysed. Percentages in parentheses represent the percentage/site per MSI or Dukes' stage group. Values of significance: ^†^*P*<0.04 and ^‡^*P*<0.03, both by *χ*^2^ test.

), Dukes' stage A or D cancers were exclusively left-sided CRC (*P*<0.03, *χ*^2^; Table 3) although the numbers were small.

Micro-satellite instability was related to gender with more MSI-H (83 *vs* 17%), and less MSI-L (33 *vs* 67%) in females than males (*P*<0.02, *χ*^2^; see Table 2), and to tumour site with MSI-H in right-sided (75 *vs* 25%) and MSI-L in left-sided (67 *vs* 33%) cancers (*P*<0.04, *χ*^2^; Table 3). Micro-satellite instability was unrelated to Dukes' stage (results not shown).

**Figure 1:**
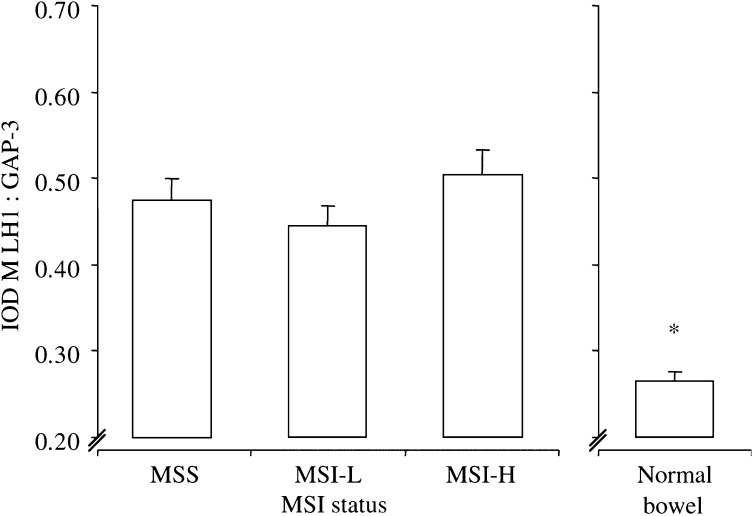
Transcription of MLH1. Transcription of MLH1 in MSI cancers and sections of normal bowel. Bars represent the mean values+s.e.m. IOD, total integrated optical density of MLH1 : GAP-3; each value represents the overall mean value for all grouped samples, derived from the mean of two separate PCR analyses per sample; see ‘Materials and methods’. ^*^P<0.01 *vs* MSS/MSI-L/MSI-H, one-way ANOVA with Bonferroni's correction.

**Figure 2:**
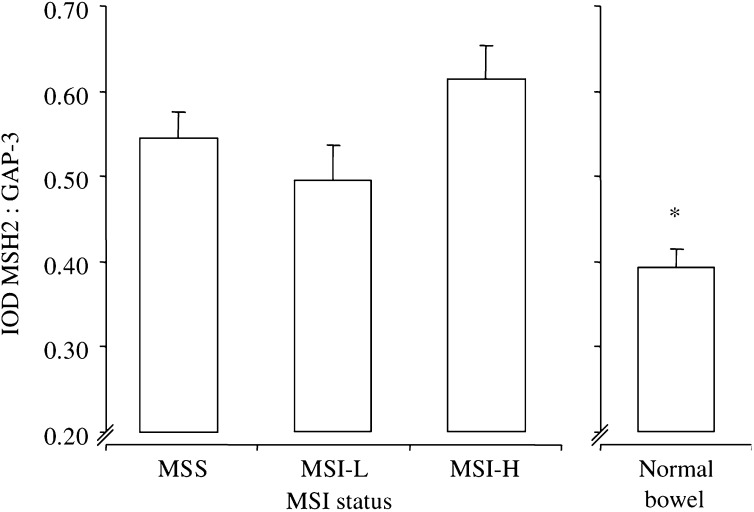
Transcription of MSH2. Transcription of MSH2 in MSI cancers and sections of normal bowel. Bars represent mean values+s.e.m. IOD, total integrated optical density of MSH2 : GAP-3; each value represents the overall mean value for all grouped samples, derived from the mean of two separate PCR analyses per sample; see ‘Materials and methods’. ^*^P<0.04 *vs* MSI-H, one-way ANOVA with Bonferroni's correction.

MLH1 transcription was similar in both MSS and MSI-L cancers, but was slightly (although not significantly) elevated in MSI-H (Figure 1

). Compared to normal bowel, all cancers demonstrated elevated levels of MLH1 transcription (all, *P*<0.01 *vs* MSS, MSI-L and MSI-H). A similar pattern of gene transcription was seen for MSH2. Although not significant, MSH2 transcription was again marginally elevated in MSI-H compared to MSS or MSI-L cancers (Figure 2

). However, MSH2 transcription was elevated only in MSI-H cancers (*P*<0.04; Figure 2) when compared to samples of normal bowel, and not in MSS or MSI-L as seen with MLH1 transcription. There was a positive correlation between MLH1 and MSH2 transcription (*P*<0.001, Pearson's bivariate correlation).

**Figure 3:**
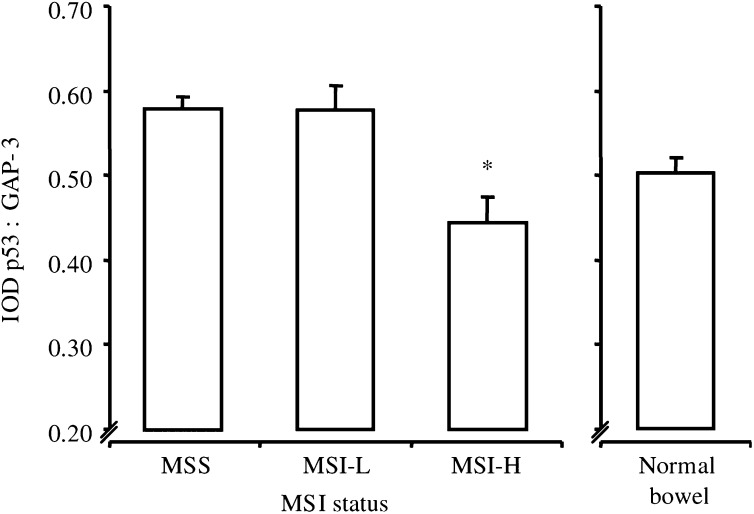
Transcription of p53. Transcription of p53 in MSI cancers and sections of normal bowel. Bars represent mean values+s.e.m. IOD, total integrated optical density of p53 : GAP-3; each value represents the overall mean value for all grouped samples, derived from the mean of two separate PCR analyses per sample; see ‘Materials and methods’. ^*^P<0.003 *vs* MSS and MSI-L, both one-way ANOVA with Bonferroni's correction.

The pattern of p53 transcription was different. Although similar in MSS and MSI-L cancers, p53 transcription was markedly reduced in MSI-H cancers (*P*<0.003 *vs* MSS and MSI-L; Figure 3

). When compared to samples of normal bowel, p53 transcription was elevated in MSS and MSI-L but reduced in MSI-H cancers, but differences were not statistically significant (Figure 3). There was an inverse correlation between MSI and p53 transcription (*P*<0.002, Pearson's bivariate correlation).

MLH1 and MSH2, and similarly p53 transcription, were not related to Dukes' stage, or site of tumour (results not shown).

**Figure 4:**
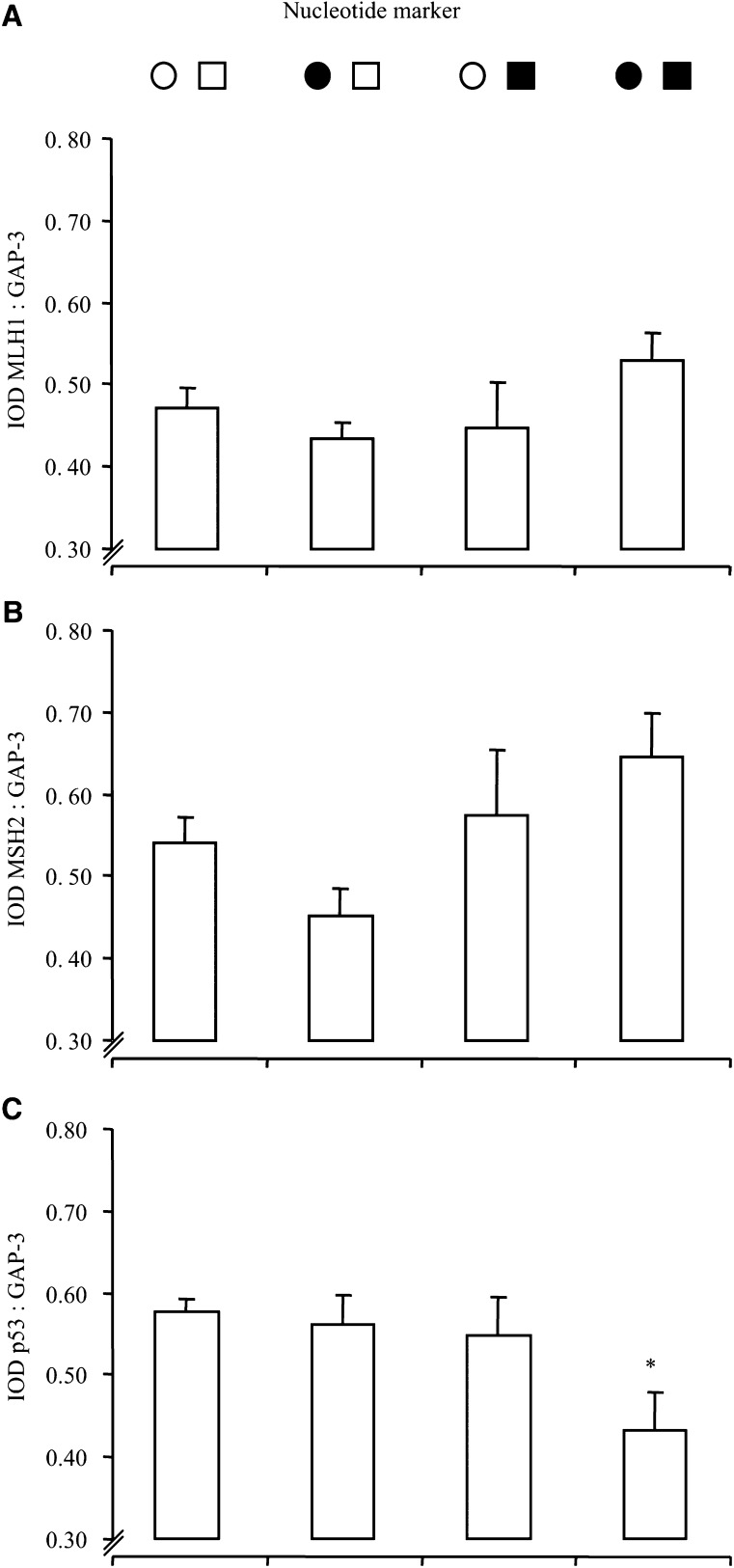
Gene transcription with nucleotide markers. Transcription of MLH1 (**A**), MSH2 (**B**) and p53 (**C**) in MSI cancers per nucleotide marker. Bars represent the mean values+s.e.m. IOD, total integrated optical density of Gene : GAP-3; each value represents the overall mean value for all grouped samples, derived from the mean of two separate PCR analyses per sample; see ‘Materials and methods’. Key to symbols: circles, mono-nucleotide markers; squares, di-nucleotide markers; open symbols, normal (non-mutated marker); closed symbols, mutated marker. ^*^P<0.01 *vs* ○□, one-way ANOVA with Bonferroni's correction. There was an inverse correlation between mutated markers and p53 transcription, *P*<0.02, Pearson's bivariate correlation.

MLH1 and MSH2 transcription was independent of mutations in mono- or di-nucleotide markers (Figure 4

). However, p53 transcription was markedly reduced when both mono- and di-nucleotide makers mutated in comparison to the normal mono- and di-nucleotides group (*P*<0.01; Figure 4C). Furthermore, increasing ‘severity’ of mutation (in mono-, in di-, then in both mono- and di-nucleotide markers) correlated with reduced transcription of p53 (*P*<0.02, Pearson's bivariate correlation; see Figure 4C).

## DISCUSSION

The discovery of MSI and the genes mediating MMR have contributed greatly to our understanding of the process(es) of tumorigenesis (Ionov *et al*, 1993; Peltomaki *et al*, 1993; Thibodeau *et al*, 1993). Studies on tumours with MSI phenotype have until recently remained restricted mainly to analyses of mutations in DNA sequences or production of protein as measured by immunohistochemistry. The importance of control of gene transcription in tumorigenesis is particularly identifiable in MSI-H cancers through silencing of gene transcription by hypermethylation of sequences important in the control of the MMR genes (Cunningham *et al*, 1998; Kuismanen *et al*, 1999). It was relevant, therefore, to investigate the levels of transcription, that is, activity, of the MMR genes MLH1 and MSH2, and the tumour-suppressor gene p53 in sporadic CRC. This was achieved by utilising wax-embedded specimens that allowed access to archival tissue for RT–PCR analyses of the genes of interest on extracted total cellular RNA.

The study sample consisted of 55% of cancers of the MSS phenotype, 27% MSI-L and 18% MSI-H, and, consistent with other reports, the MSI-H cancers were predominant in females and in the right colon (Boland *et al*, 1998; Thibodeau *et al*, 1998).

The prevalence of MSI-H in sporadic CRC has drawn attention to methylation of MLH1 promoter regions as the potential mechanism underlying MSI in these cancers (Cunningham *et al*, 1998; Herman *et al*, 1998). Accordingly, in the specimens examined, levels of MLH1 transcription were expected to be reduced. However, MLH1 transcription was higher in all cancers than in normal bowel, and, contrary to other studies (Kane *et al*, 1997; Cunningham *et al*, 1998; Herman *et al*, 1998; Kuismanen *et al*, 1999), was increased in MSI-H compared to MSS and MSI-L cancers. Although contrary to current theories of gene expression downregulation by methylation in MSI-H cancers (Cunningham *et al*, 1998; Herman *et al*, 1998), a possible explanation for this finding may be that the total cellular RNA extracted from the paraffin-embedded samples might have included adjacent normal tissue that expressed MLH1. Furthermore, should methylation occur at only one allele (hemi-methylation), transcription could still occur from the other nonmethylated allele (Cunningham *et al*, 1998; Kuismanen *et al*, 1999). However, as MLH1 mRNA was amplified, it must have been available for translation to protein, although on immunohistochemistry these cancers demonstrate reduced MLH1 protein expression (Kane *et al*, 1997; Cunningham *et al*, 1998; Herman *et al*, 1998; Kuismanen *et al*, 1999). Reduced translation from mRNA to protein, or post-translational modifications of MLH1 rendering it less stable or with a reduced half-life might explain these findings. Alternative mechanisms other than DNA methylation might also be responsible, for example, the mutation of genes such as MSH3 (Risinger *et al*, 1996), or polymerase delta (da Costa *et al*, 1995). As 70–80% of MSI-H cancers appear to be related to the epigenetic inactivation of MLH1 (Cunningham *et al*, 1998; Herman *et al*, 1998), in our samples, the production of amplifiable mRNA for MLH1 suggests a post-transcriptional mechanism as the basis for reduced MLH1 function in these cancers. The pattern of MSH2 transcription was overall similar to that of MLH1 and, accordingly, similar factors might be involved. The direct association between increased transcription of MLH1 and MSH2 with MSI might suggest that their activity is at least in part co-ordinated, perhaps as an attempt to control the higher mutation rate in MSI-H.

An intriguing finding was the reduction in p53 transcription in MSI-H compared to normal bowel, and MSS or MSI-L cancers. While studies on p53 mutation in MSI have shown conflicting results, there is a consensus that p53 has an inverse relationship with MSI (Cottu *et al*, 1996; Breivik *et al*, 1997; Olschwang *et al*, 1997; Forster *et al*, 1998; Iacopetta *et al*, 1998). The basis for the apparent reduced rate of p53 mutations in the higher ‘mutator’ phenotype of MSI-H has remained unclear. Immunohistochemistry is widely used to identify p53 mutation, with overexpression being regarded as a measure of mutation of the gene (Rodrigues *et al*, 1990). However, the results from different studies should be interpreted with caution, taking into consideration the processes of tissue fixation and staining, the antibodies employed and the scoring technique adopted. For example, differences in reported rates of p53 mutation can be accounted for by the inconsistent measurement of stained cells that varied from 5 to 30% in determining if a cancer is mutated or not (Kim *et al*, 1994; Ilyas *et al*, 1996; Iacopetta *et al*, 1998). Moreover, p53 overexpression can occur in the absence of mutation due to the accumulation of wild-type p53 in cancer cells (Cripps *et al*, 1994; Dix *et al*, 1994), again highlighting the potential for mis-assigning p53 mutations in MSI cancers if mutation is based on overexpression of p53 protein. Alternatively, p53 may indeed be mutated, but remains largely undetected as the resultant protein is truncated and so unligated by the detecting antibody (Cripps *et al*, 1994; Dix *et al*, 1994). Direct sequencing and SSCP also have their limitations in detecting p53 mutations. With direct DNA sequencing, contamination from normal DNA can mask the true result of tumour DNA analysis, and although SSCP has been widely used for screening mutations these have been mainly in exons 5–8 of p53 (Iacopetta, 2003), but not other regions. In our samples, a reduced rate of p53 transcription to a level below that in normal bowel might explain the reduced p53 function in these cancers. In addition and of equal importance is that a reduced rate of p53 transcription would help explain the apparent reduction of p53 mutations in MSI-H cancers when this has been determined by the criteria of overexpression of protein. Here, the reduced rate of transcription predisposes reduced protein production, and, as fewer cells are detected immunohistochemically as expressing p53, consequently the rate of mutation judged by p53 overexpression is also reduced.

There is evidence that epigenetics plays an important role in tumorigenesis. Toyota *et al* (1999) describe the CpG island methylator phenotype (CIMP) to denote widespread methylation of several genes implicated in the tumorigenic process(es). Recently, methylation of p14 was observed in MSI-H cancers that were CIMP positive and with fewer p53 mutations (Shen *et al*, 2003), although previously p14 promoter hypermethylation was reported to be only marginally related to p53 mutation (Esteller *et al*, 2000). p14 is a cell cycle regulatory protein known to interact *in vivo* with MDM2, resulting in a decreased MDM2-mediated degradation of p53 (Stott *et al*, 1998). Theoretically, as an upstream regulator of p53 (via reducing degradation), when p14 is inactivated via methylation, the resultant increase in MDM2 would in turn reduce the availability of p53 and so potential tumour suppression. Hence, methylation of p14 might obviate the need for p53 mutation in tumorigenesis. The principle regulation of p53 is by post-translational modification of the protein rather than at the transcriptional level of the gene (Oren, 1999). However, the difference in transcription of p53 between MSI-H and MSS/MSI-L cancers in this study might suggest a role in MSI.

Cancer location has been associated with genetic differences (Delattre *et al*, 1989; Gervaz *et al*, 2001). Infrequent loss of heterozygosity (Delattre *et al*, 1989) and fewer p53 mutations (Breivik *et al*, 1997) have been more commonly associated with right-sided cancers. However, in this series, there was no difference in the levels of gene transcription between cancers of the left or right colon. Differences in the micro-environment of the colon, or factors other than MLH1, MSH2 or p53 transcription, are more likely to be involved. Transcription of MLH1 and MSH2 was also independent of Dukes' stage.

The NCI guidelines propose examination of two mono-nucleotide and three di-nucleotide markers in order to classify the MSI state (Boland *et al*, 1998). Mono-nucleotide markers are relatively stable, nonpolymorphic, and are sensitive for the MSI-H phenotype (Ionov *et al*, 1993; Dietmaier *et al*, 1997), while instability in MSI-L is essentially restricted to di-nucleotide (or longer) repeat markers (Dietmaier *et al*, 1997). A potential problem arises, however, when there is a singular mutated mono-nucleotide marker. In these cases, although such mutations are sensitive for MSI-H (Ionov *et al*, 1993; Dietmaier *et al*, 1997; and so may reflect an MSI-H-associated underlying mechanism), the singular mutated marker would result in the MSI-L classification. This was examined more closely by analysing the levels of gene transcription in terms of MSI as well as mutations in mono- or di-nucleotide markers.

Transcription of MLH1 and MSH2 was overall largely unaffected by particular nucleotide mutations (see Figure 4), although there was a slight, nonsignificant trend for the levels of transcription to increase with increasing numbers of mutations. In contrast, increasing ‘severity’ and number of mutations (in mono-, in di-, then in both mono- and di-nucleotide markers) correlated overall with reduced transcription of p53 (*P*<0.02; see Figure 4C). However, it is important to note here that these tumours reflect a large proportion of the MSI-H subgroup and therefore overall with reduced p53 transcription (see Figure 3).

In conclusion, RT–PCR analysis of wax-embedded samples is both feasible and of value. Of greater importance, however, is that the reduced transcription of p53 in MSI-H emphasises a molecular difference in this subgroup of cancers, in which the investigation of molecules such as p14 and MDM2 in these cancers would help clarify the role of p53 in MSI.
